# Building resilience to climate change: designing research with health system and community perspectives

**DOI:** 10.1177/17579139251365576

**Published:** 2025-09-12

**Authors:** JG Richmond, P Beltran-Alvarez

**Affiliations:** Centre for Healthcare Innovation, Policy and Management, School of Business, University of Leicester, University Road, Leicester, UK; Biomedical Institute for Multimorbidity, Centre for Biomedicine, Hull York Medical School, University of Hull, UK

## Abstract

This feature is a call to action for researchers: RIchmond and Beltran-Alvarez provide important insight to researchers in the area of climate change, public health, and health systems. The article details how they can design research which integrates perspectives of both community and health system level stakeholders to ensure that any strategies they implement consider the intersection between these important groups.

As a result of climate change, extreme weather events (EWE) such as flooding, sea level rise and associated storm surges, and extreme heat are more frequent, intense, widespread and costly. EWE result in negative impacts on health systems and the populations they serve, including driving hospital admissions and lengths of stay, overheating and flooding facilities, and worsening symptoms for people with existing health conditions, including mental health.^1,2^

Among the recent policy implementations undertaken by the UK’s National Health System, achieving a net zero operation by 2040 is prioritised by the national government as a top-down policy imperative.^3^ As such, the National Health Service (NHS) trusts, who, across England, struggle with everyday operational pressures,^4^ are tasked to implement actions to mitigate emission of the greenhouse gases that drive climate change. This is in addition to leading emergency preparedness responses and resilience-focused adaptations for EWE, which minimise hazards and impacts on healthcare systems and human health.^5^

Effectively adapting to and mitigating EWE presents a major resource challenge for the NHS, leaving less capacity for bottom-up community engagement which can help make in-roads and build trust with vulnerable and ethnic minority populations. This often overlooked aspect is critical because EWE do not affect populations equally.^6^ The COVID-19 pandemic highlighted that disadvantaged communities and those underserved by formal public health systems are among the most vulnerable to the effects of systemic shocks and extreme events. Climate vulnerability drivers, such as deprivation and social isolation, also underlie poor wellbeing and health inequalities. Climate change therefore has the potential to widen existing health inequalities.^7^

**Figure:**
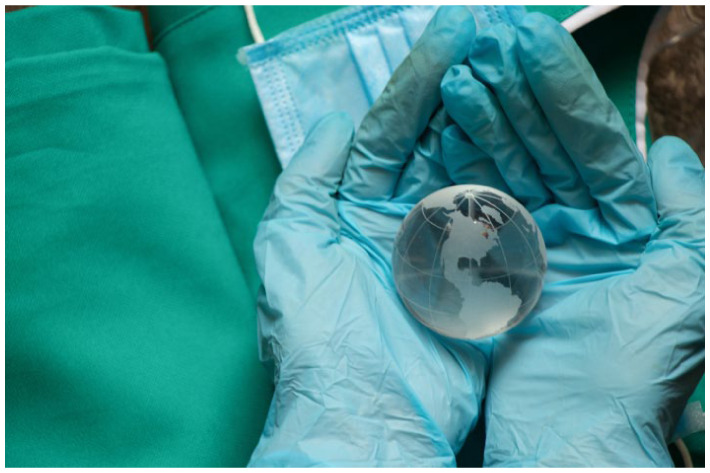


While there are clear UK objectives for addressing climate change, net zero and health outcomes, a health equity and climate justice lens is notably lacking from policy development work, which may inadvertently lead to further health disparity.^8^ For example, poorly considered climate change interventions may lead to co-harms (health trade-offs or intervention-generated inequalities^9^) and prevent the full realisation of the health co-benefits of transitioning to net zero. Research finds that current interventions for EWE, such as heat-health risk messaging, do not adequately consider health equity and provide little evidence of benefit to the health service, particularly when considering access by vulnerable groups.^10,11^

We highlight that a multi-layered and multi-scalar approach is required to address these issues. Future research must contribute to practice by addressing the need for a ‘one health’ approach to the design and implementation of all policy that is system-wide, from the ‘top-down’ and ‘bottom-up’, in order to reduce vulnerability and build resilience into society, organisations and systems, while protecting the health of people and the planet.^12^

Identifying vulnerability is multi-faceted and challenging. Some populations are inherently vulnerable, while others are transiently vulnerable due to the sporadic and intense nature of EWE and the impact they have on an individual’s environment and wellbeing.^6^ There are likely to be additional groups that are specifically vulnerable to EWE which are currently unidentified in a UK population (e.g. healthy adults who work outside, school children, those with mental illness). As such, where multiple axes of vulnerability are present an intersectional approach is warranted.

Recent studies^11,13,14^ call for more research to examine whether, how, and which interventions for EWE are effective for public health. They flag that current adaptations require better coordination between organisations, do not adequately consider health equity, and are generally insufficient.^14^ This requires system-level working across government, national, and local public sector bodies and local authorities. For example, the Institute for Health Equity report^8^ recommends an overarching health-equity-in-all-policies approach, which considers how decisions made in all government departments have implications for health, health equity, and climate change.

System resilience to climate change is more nuanced than individual risks, due to the interconnectedness of all infrastructure sectors. Assessing climate risks in systems and organisations is complicated by multiple, interacting risks. Therefore, vulnerabilities on one network or organisation can lead to a wider-ranging impact across sectors as issues ‘cascade’ beyond the primary infrastructure. The third iteration of the National Adaptation Programme (NAP3) cautions that, to-date, the vulnerability of interconnected systems may be significantly underestimated;^15^ hence, recent public health resilience research calls for a whole systems approach to managing EWE.^13^

Considering these challenges, we recommend that future research in this area is designed to address the following opportunities:

First, consider an overarching system-focused, place-based approach, which embeds community and stakeholder engagement.^16^ This includes a complex, adaptive systems perspective which enables consideration of interventions for EWE amid a complex health and societal system that necessitates implementation and evaluation at different levels. This approach will help understand the interaction between interventions for EWE and the context in which they are implemented in a dynamic way. We suggest researchers seek input from stakeholders, including those who develop the relevant policies, those who are targeted by them, and others who are responsible for operationalising plans for EWE.

Second, from the ‘top-down’ perspective, consider how health systems cope with and adapt to the challenge of EWE, and the possible synergies or overlap between mitigation and adaptation strategies (e.g. urban green spaces). From the ‘bottom-up’, establish co-production research with communities to understand emergent interventions for EWE, specifically, to ask how communities are adapting to extreme weather; how emergent community responses resonate with top-down decisions, and if any intervention-generated inequalities exist.

Third, compare and contrast the existing literature with the live experiences of community stakeholders to understand the nuances of vulnerability and which characteristics make people more exposed, and more vulnerable to EWE. This should include developing an understanding of the characteristics of populations most vulnerable to the health impacts of EWE.

Finally, ensure that research outputs, including policy and practice implications are relevant and actionable by stakeholders at both the health system and community level. We recommend following a co-design approach including the use of ‘living labs’, defined as spaces of sustainability experimentation that are characterised by geographical embeddedness in a specific context, an explicit learning function, participation, and user involvement.^17^
